# Fundus albipunctatus: novel mutations and phenotypic description of Israeli patients

**Published:** 2012-06-23

**Authors:** Eran Pras, Elon Pras, Haike Reznik-Wolf, Dror Sharon, Svetlana Raivech, Yaniv Barkana, Almogit Abu-Horowitz, Rotenstreich Ygal, Eyal Banin

**Affiliations:** 1 Department of Ophthalmology, Assaf Harofeh Medical Center, Zerifin, Israel; 2 Gartner Institute of Human Genetics, Sheba Medical Center, Tel Hashomer, Israel; 3 Department of Ophthalmology, Hadassah-Hebrew University Medical Center, Jerusalem, Israel; 4 Electrophysiology Clinic, Goldschleger Eye Research Institute, Sheba Medical Center, Tel Hashomer, Israel; 5 Sackler School of Medicine, Tel Aviv University, Tel Aviv, Israel

## Abstract

**Purpose:**

To characterize the genetic defects associated with fundus albipunctatus (FAP) in patients in Israel.

**Methods:**

Twenty patients with FAP from diverse ethnicities underwent ophthalmic and electroretinogram tests following the International Society for Clinical Electrophysiology of Vision protocol. Genomic DNA was extracted from peripheral blood. Mutation analysis of the 11-cis retinol dehydrogenase (*RDH5*) gene was performed with direct sequencing of PCR-amplified exons.

**Results:**

Four novel *RDH5* gene mutations were identified. Of them, the null mutations c.343C>T (p.R54X) and c.242delTGCC were most prevalent. Macular involvement was present in two patients who carry different mutation types.

**Conclusions:**

Mutation analysis of the *RDH5* gene in the present series revealed four novel mutations and a previously reported one. No significant genotype-phenotype correlation was found.

## Introduction

Fundus albipunctatus (FAP; OMIM 136880) is a relatively mild and rare form of congenital stationary night blindness (CSNB) with a characteristic appearance of numerous small, subretinal, white-yellow spots in the perimacular area and the retinal periphery. FAP is an autosomal recessive disease caused almost exclusively by mutations in the 11-cis retinol dehydrogenase (*RDH5*) gene, which is expressed predominantly in the retinal pigment epithelium, and encodes for 11-cis retinol dehydrogenase [1]. This enzyme participates in the retinoid cycle that regenerates the visual chromophore 11-cis-retinaldehyde (11-cis-RAL) from all-trans-retinaldehyde (all-trans-RAL), which is then transported to the photoreceptors for integration into rhodopsin and cone opsins, making them amenable for activation by light [2]. The final step in this enzymatic cycle involves the oxidation of 11-cis-retinol to 11-cis-RAL by *RDH5*. Recently, FAP has been observed in a compound heterozygous mutation carrier of another retinoid cycle gene: *RPE65* [3]. Patients with *RDH5* mutations experience extremely long dark adaptation times due to the deficient 11-cis chromophore in rod photoreceptors. The characteristic white retinal spots in this disorder, which presumably represent local accumulations of retinoids due to the loss of *RDH5* function [4], spare the macular region. Therefore, FAP has traditionally been regarded as an exceptionally mild retinal dystrophy that does not markedly affect central vision, as opposed to defects in nearly all other steps of the visual cycle that often manifest with widespread, severe, and often early retinal degeneration. However, subsequent studies have shown in a subset of patients that the disease is not stationary: cone visual function deteriorates, and cone dystrophy may later develop [5-13]. Liden et al. have suggested that cone degeneration may be a direct consequence of a decreased supply of 11-cis retinal to the cones [14]. Recent estimates suggest that the prevalence of cone dysfunction is higher than previously appreciated and can affect more than one third of patients [12,15]. Significant progress in understanding the pathogenesis of FAP arose through the study of *Rdh5* knockout mice models [4,16]. These models provide evidence for the existence of alternative oxidative pathways that bypass *RDH5* activity and may explain why night vision regenerates after prolonged dark adaptation even in the absence of *RDH5* activity [17]. Moreover, pharmacologic treatment with 9-cis-retinal was shown to have benefits in an *Rdh5* knockout mice model [18], and improvement by 9-cis β-carotene in dark adaptation was recently demonstrated in humans with *RDH5* mutations [19]. When faced with a young patient manifesting white-yellow retinal spots and night blindness, three main clinical diagnoses may be considered: FAP with the characteristic, relatively favorable CSNB phenotype, FAP that will progress to include macular involvement, or, alternatively, the much more severe widespread retinal degeneration of retinitis punctata albescens (RPA). Electrophysiological examinations have the potential to distinguish among these phenotypes, mainly by demonstrating the typical delayed dark-adapted recovery of the scotopic electroretinogram (ERG) in FAP versus the lack of recovery in RPA. The scotopic rod and mixed cone-rod full-field ERG responses recorded after the normal 30–45 min of dark adaptation are severely attenuated in both conditions. However, repeating the recording after prolonged dark adaptation (>3 h) reveals significant recovery in FAP while it remains markedly reduced in RPA [1]. In recent years, molecular genetic studies have allowed further improvements in verifying a diagnosis of FAP, by demonstrating mutations in the *RDH5* gene. To date, 34 *RDH5* mutations have been reported. Most are missense mutations; however, a few frameshift and in-frame mutations have been reported [6,15,20]. Here, we report four novel *RDH5* mutations identified in a large cohort of Israeli patients with FAP.

## Methods

The study was approved by the Assaf Harofeh Medical Center institutional review board, and informed consent that adhered to the tenets of the Declaration of Helsinki was obtained from all participants. Initially, we conducted a search for patients with FAP by reviewing medical records and electrophysiology examinations at three ophthalmic centers in Israel: Hadassah-Hebrew University Medical Center, Jerusalem; Sheba Medical Center, Tel Hashomer; and Assaf Harofeh Medical Center, Zerifin. The following data were obtained: age, ethnic background, family history, best-corrected visual acuity, slit lamp biomicroscopy, fundus examination, fundus photography, color vision testing using the Ishihara and Farnsworth D-15 tests, and full-field ERG recordings as previously described [21]. Briefly, full-field ERGs were recorded using Bipolar Burian Allen corneal electrodes (Henkes-type, Medical Workshop B.V., Groningen, the Netherlands) and a computerized system (UTAS 3000; LKC, Gaithersburg, MD). Cone responses to 30-Hz flashes of white light were acquired under a background light of 21 cd/m^2^. Scotopic responses including a rod response to a dim blue flash and a mixed cone-rod response to a white flash were initially acquired following 30–45 min of dark adaptation. Afterwards, one eye was patched, and scotopic responses were recorded again from this eye after at least 3 h of dark adaptation. Between two and four sets of responses were recorded in each condition to verify repeatability. All ERG responses were filtered at 0.3 to 500 Hz, and signal averaging was used. In all examined patients except one (MOL 0091–06, who refused sampling of his blood), DNA was extracted from peripheral blood using a commercial kit (Gentra System Inc., Minneapolis, MN). One hundred subjects without ocular disease were used as controls. The 5′ untranslated region (UTR) of the *RDH5* gene and all exons and their boundaries were amplified and sequenced as previously described [22]. Briefly, polymerase chain reaction (PCR) amplification was performed using three sets of the following primers:

For exon 1 and 5′UTR region: Fw. 5′-CTT GGA GAC CAT GAC ATA GAT GG-3′; Rw. 5′-GTA TCA CAC AGC AAA CCT CTT GG-3′, for exons 2–3 Fw. 5′-ATA TGC TCA CAC CAG ATG CTT CC-3′; Rw. 5′-ACT CAA CAA ACG TGG GCC AG-3′, and for exons 4–5 Fw. 5′-AAC CCA TGT CCC TCA AAG TCC-3′; Rw. 5′-CAA CTA CTC CAA ACC GAC ATG CT-3′. The reaction took place in a 25 μl volume containing 50 ng of DNA, 13.4 ng of each primer, 1.5 mM dNTP’s, in 1.5 mM MgCl_2_, PCR buffer, with 1.2 U of Taq polymerase (Bio-Line, London). After an initial denaturation of 5 min at 95 °, 30 cycles were performed (94 ° for 2 min, 57 ° for 3 min and 72 ° for 1 min), followed by a final extension of 7 min at 72 °. PCR amplicons were sequenced in both directions using a commercial sequencing service (Hy Laboratories, Ltd. (Hylabs) Park Tamar Rehovot, Israel).

## Results

**Figure 1:**
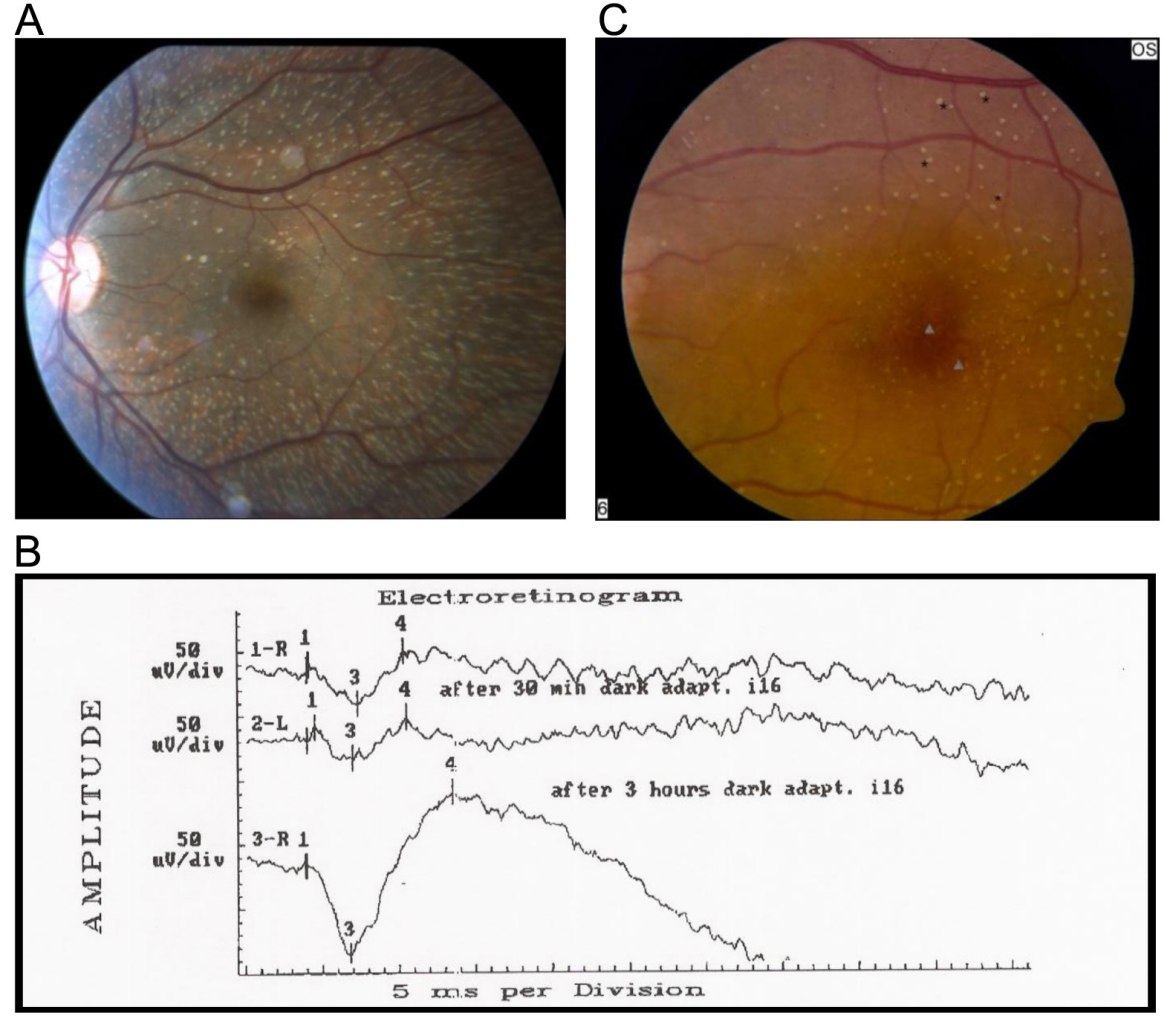
Clinical findings in fundus albipunctatus patients. **A**: Fundus photograph of the left eye of patient MOL0427–1 at the age of 19 years showing multiple small white-yellow retinal spots and flecks with relative sparing of the fovea. **B**: ERG recordings of patient MOL0427–1 under scotopic conditions showing markedly reduced mixed cone-rod responses after 30 min of dark adaptation in both eyes. Following 3 h of dark adaptation in the right eye, the responses significantly improved and reached the normal range. **C**: Fundus photograph of the left eye of patient MOL0091–2 at 29 years of age. Small patches of yellowish atrophy at the fovea are apparent (arrowheads) in addition to preexisting punctata (asterisks).

We identified 20 patients with FAP from 14 families (Table 1), based on the following criteria: a history of poor night vision with prolonged dark adaptation, presence of white-yellow retinal spots on ophthalmoscopy (Figure 1A), and the demonstration of recovery of scotopic full-field ERG responses following prolonged dark adaptation (Figure 1B) [21]. Thirteen families were of Jewish descent, and one, which included three patients, was of Arab-Muslim origin. In 11 of the families, only one affected individual was identified, Table 1.

**Table 1:** Demographic, clinical and genetic findings of patients with fundus-albipunctatus.

**Family**	**Patient**	**Ethnicity**	**Age at exam**	**BCVA OD:OS**	Cone Dysfunction/ Maculopathy*	**Mutations**
14	1	Buchara	35, 38	20/20: 20/20	-	R54X /R54X
	2		40	20/20: 20/20	-	R54X /R54X
15	1	Iraq/Morocco	50	20/25: 20/25	-	R54X /R54X
19	1	Iran	32	20/25: 20/20	-	R54X /R54X
17	1	Iraq	50	20/25: 20/25	-	R54X /R54X
	2		39	20/20: 20/20	-	R54X /R54X
	3		55	20/20: 20/30	-	R54X /R54X
18	1	Iraq	32	20/20: 20/20	-	R54X /R54X
MOL0427	1	Iran	19	20/20:20/20	-	R54X /R54X
MOL0580	1	Iraq	55	20/50: 20/70	M.A.; Tr.; ↓30Hz	R54X /R54X
16	1	Ashkenazi	29, 31	20/25: 20/25	-	c.242delTGCC/ c.242delTGCC
20	1	Iraq/Ashkenazi	22	20/20: 20/20	-	c.242delTGCC/ c.242delTGCC
MOL0338	1	Ashkenazi	23	20/20: 20/20	-	c.242delTGCC/ c.242delTGCC
MOL0091	2	Arab-Muslim	18	20/20: 20/20	M.A.; ↓30Hz	D128N/D128N
			29	20/50: 20/40		
			35	20/200:20/400		
	3		10	20/30: 20/30	-	D128N/D128N
	4		23	20/30: 20/30	-	D128N/D128N
	6		24	20/20: 20/20	-	NO DNA
21	1	Turkey/Iraq	20	20/20: 20/20	-	R191Q/_____
22	1	Morocco/India	24	20/20: 20/25	-	R278Q/_____
23	1	Yemen	55, 69	20/30: 20/25	-	——-/——

Ethnicity: when both parents are of the same origin, it is listed; when of different origins, presented as paternal/maternal. All families except MOL0091 were of Jewish descent. Age at exams: When more than one examination was performed it is listed in the same row if visual acuities did not change and in consecutive rows if vision has deteriorated. Visual acuities: Best corrected visual acuities (BCVA) were 20/30 or better in all patients except MOL0580–1 and MOL0091–2 whose visual loss was gradual and documented longitudinally in repeated exams. Mutations: when mutations were identified in both alleles it is indicated as mutation/mutation, when a mutation was identified only in one allele: mutation/______, and when no mutations were found it is shown as dashes only: _____/______. *Cone dysfunction/Maculopathy: The following abbreviations are used in description: “M.A” for macular atrophy, “Tr.” for tritanopia, and “↓30Hz” for reduced cone flicker ERG.

### Clinical findings

All recruited patients manifested the characteristic widespread subretinal white-yellow spots on funduscopy, and ERG testing showed an initial severe impaired scotopic response that markedly improved following prolonged dark adaptation (Figure 1). Visual acuities ranged from 20/20 to 20/30 in all patients except MOL0580–1 (at age 55) and MOL0091–2 (at age 18) who also manifested macular atrophy, Table 1. Patient MOL0580–1 noted that her visual acuity in the past had been much better and that the decrease in vision had occurred gradually over the last few years. Patient MOL0091–2 was noted to have night blindness at early childhood, and progressive deterioration of central visual function began in adolescence. On repeated ERG exams, cone flicker responses gradually deteriorated, with reduced amplitudes and delayed implicit times. At the age of 35 years, he became almost legally blind, with low visual acuities of 20/200 in his right eye and 20/400 in the left eye. Funduscopy revealed the characteristic widespread subretinal white spots, accompanied by macular atrophy. Three of his sisters, MOL091–03, MOL091–04, and MOL091–6, manifested typical FAP findings without macular involvement at the age of 10, 23, and 24 years, respectively, and on repeated examinations during the last two decades.

### Molecular studies

**Figure 2:**
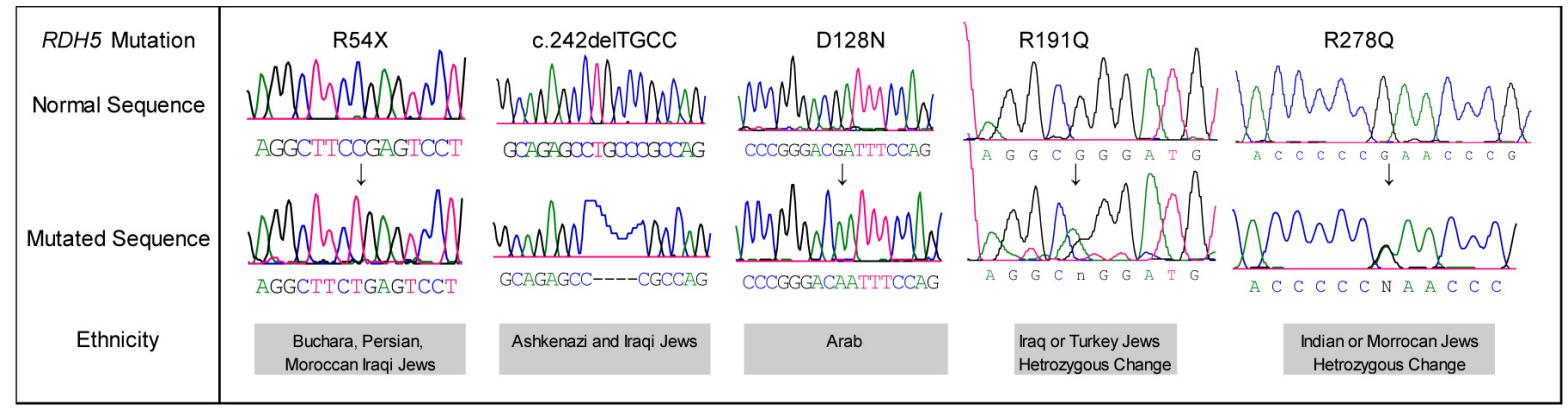
*RDH5* mutations identified in this study. Partial *RDH5* sequences of unaffected control (upper row) and patients with FAP (bottom row) are shown.

*RDH5* mutations were found in all patients except one (23–01; Table 1 and Figure 2). The previously reported missense c.565 G>A transition mutation (p.D128N) [23] was identified homozygously in one Arab-Muslim family (MOL0091), while all other mutations were novel and observed in Jewish patients of diverse ethnicities. The c.343C>T mutation results in the formation of a stop codon at position 54 (p.R54X) in the encoded protein, and c.242delTGCC causes a frame-shift starting at codon 24 with a putative stop codon 47 amino acids downstream. The p.R54X mutation was found in the homozygous state in ten patients, including five sporadic patients and five siblings belonging to families 014 and 017 (Table 1). Notably, p.R54X was found solely in Jews from neighboring Asian countries including Iran, Uzbekistan, and Iraq, while c.242delTGCC was present in Ashkenazi and Iraqi Jews. Interestingly, deleterious mutations (R54X and c.242delTGCC) were relatively common in our cohort compared to previous descriptions [6,15,20].

Surprisingly, even though FAP is considered an autosomal recessive disease, in two patients only a single heterozygous mutation was identified; c.755 G>A (p.R191Q) and c.1016 G>A (p.R278Q; Table 1 and Figure 2). Sequence alignment of the 11-cis-retinol dehydrogenase with its orthologs at the positions of the corresponding amino acids revealed that arginine 191 and 278 are conserved throughout evolution. Additional sequencing effort was made in these DNA samples by analyzing the 5′ untranslated region of the gene, but no other sequence alterations were demonstrated. We were unable to recruit additional family members of these patients; however, the patients reported that none of their relatives have night vision disturbances. None of the five mutations found in our patients with FAP were detected in a panel of 100 ethnically matched controls.

## Discussion

The present survey expands the spectrum of *RDH5* mutations causing the FAP phenotype by identifying four novel mutations. With direct sequencing, we identified mutations in 34 of 38 (89%) carrier chromosomes. Two mutations were identified in 16 of the 19 patients, a single heterozygote mutation was identified in two patients, and in one patient no mutations were identified at all. Despite the high mutation detection rate in this study, 10% of the carrier chromosomes remained uncharacterized for which there are three potential explanations. First, some mutations may have escaped detection, i.e., exon deletions, large chromosomal rearrangements, or mutations in the introns and promoter regions that were not analyzed. Second, in the two patients with a single heterozygous mutation we cannot rule out autosomal dominant inheritance with reduced penetrance. According to functional essays, specific heterozygous *RDH5* mutations can interfere with the function of a second normal allele, and exert a negative-dominant effect [14]. Finally, although FAP has been considered a monogenic disease until recently, growing evidence implicates other retinoid cycle genes that may act alone or interact with *RDH5* itself in disease pathogenesis [3].

Two of the five mutations were identified in ten out of the 14 studied families (71%), in populations that have previously shown founder effects in other genes (see Tables of Mendelian disorders among Jews at the Israeli Ministry of Health database). R54X was detected only in Jews from central Asian countries (Iran, Iraq, and Uzbekistan) whereas c.242delTGCC was found in Ashkenazi carrier chromosomes and in one Iraqi carrier chromosome (MOL020–01), a fact that might be explained by a genetic drift or a mutational hot spot. Both mutations are expected to result in a non-functional protein or in nonsense-mediated decay of the mRNA [24]. Despite having no functional protein, most of these patients exhibit only a mild phenotype with stationary night blindness that recovers after prolonged dark adaptation. This phenomenon can be attributed to alternative, yet less efficient pathway(s) for producing the 11-cis-retinal needed for rhodopsin regeneration [16].

Cone dysfunction has developed in two patients of different ethnicities who carry different *RDH5* mutation types. One Arab-Muslim patient homozygous to the missense D128N mutation developed cone dysfunction at adolescence, whereas a more gradual development of cone dysfunction was observed in a middle-aged Iraqi Jewish patient homozygous to the null mutation R54X. The observed difference in age of onset of this untoward complication could potentially be attributed to the type of mutation and its impact on the 11-cis retinol dehydrogenase activity; however, we included in our series patients with the same mutations whose disease was stationary without cone dystrophy. Therefore, mutation type by itself does not solely determine this severe complication. Previous descriptions provide only a few examples of null *RDH5* mutations in patients with FAP [6,15,20]. The present study however, contributes 13 new cases of biallelic truncating mutations, and when their related phenotype is placed in the context of the literature, it is still impossible to provide significant phenotype-genotype correlations. Elusive modifiers that determine FAP prognosis remain to be determined in the future.
